# Multimodal Analgesia in Pediatric Cancer Pain Management: A Retrospective Single-Center Study

**DOI:** 10.7759/cureus.45223

**Published:** 2023-09-14

**Authors:** Mesut Bakır, Şebnem Rumeli, Argun Pire

**Affiliations:** 1 Pain Management, Mersin City Education and Research Hospital, Mersin, TUR; 2 Anesthesiology and Reanimation, Mersin University Faculty of Medicine, Mersin, TUR; 3 Anesthesiology and Reanimation, Mersin Tarsus State Hospital, Mersin, TUR

**Keywords:** pediatric cancer pain management, neuropathic pain, multimodal approach, interventional management, adjuvant therapy

## Abstract

Objectives

A multimodal approach to pain management, including potential interventional techniques, is suggested to achieve adequate pain control. This study discusses the techniques and medications employed to manage pain in pediatric oncology patients.

Methodology

This study included 90 patients under 18 years of age who underwent pain management in the algology clinic between 2002 and 2020. From the algology follow-up records, the following data were recorded: demographic information, follow-up time, cancer diagnosis and stage, cause and location of pain, systems involved, duration and intensity of pain, analgesic and adjuvant drugs prescribed, routes and duration of drug administration, complications, interventional procedures if performed, “pain intensity” scores prior to and following treatment, and daily and total analgesic consumption of the patients.

Results

The mean age was 11.4±4.1 years (min-max: 2-17). Leukemia and lymphoma were the most frequently diagnosed (30%). Of the 31 features identified in the staging, 27 (87.1%) were stage 4 at admission. The causes of pain in children were neoplasms in 81.2% (*n* = 73). At admission, 72.3% (*n* = 65) had severe pain for at least a month. It was determined that 90% (*n* = 81) of the patients were using opioids and 28.9% (*n* = 26) were using dual opioids. The mean tramadol dose was 129.0±97.9 mg/day (12-380 mg/day), and the mean morphine dose was 14.8±11.3 mg/day (1-52 mg/day). The mean transdermal fentanyl dose was 33.2±21.6 µgr/h (12-75 µgr/h). Adjuvant therapy was administered in 25.6% (*n* = 24) of the patients. Epidural catheterization was performed on 6.6% (*n* = 6) of the patients. The mean initial pain scores were 5.2±1.7, which decreased to 1.5±0.7 with a significant difference (*p* < 0.001). In the study, 93% (*n* = 84) of the patients had no pain management complications noted.

Conclusions

The pain level that pediatric cancer patients endure critically influences their and their family’s quality of life. The fact that opioid-related adverse effects associated with pediatric pain management occur far less frequently than previously thought may help prevent opiophobia. Effective and safe analgesia can be provided with multimodal analgesia to manage pediatric cancer pain.

## Introduction

Around 400,000 children are diagnosed with cancer annually. The World Health Organization (WHO) has started the CureAll framework study for children with pediatric cancer who are considered undertreated. One of the most vital activities of this organization is pain management [1], as only 40% cases of pediatric cancer pain are reported to be managed well [2].

Pain in pediatric cancer may be caused directly by the tumoral tissue, or it may develop due to the paraneoplastic effects of the tumor and the compression of the surrounding healthy tissue [3]. Moreover, side effects of drugs, operations, radiotherapy applications, and repeated small painful interventions also cause pain [4]. The development of pain due to many factors makes multimodal analgesia necessary [5]. In recent years, serious studies have been conducted on the applicability of the step therapy published by WHO to children [6]. However, uncertainties remain about adjuvant drugs and opioid doses [7].

Multimodal analgesia is a pain management approach that combines medicines with multiple mechanisms of action and advantageous adverse-effect profiles. Pain management that spares opioids can be effectively achieved by combining multimodal analgesia with regional anesthesia [8].

At Mersin University Algology Clinic (the medical treatment of pain practiced in Turkey is called algology), researchers have followed pediatric oncological patients for 20 years. Based on the WHO step therapy, pain palliation is provided using multimodal analgesia. This study aims to share the details of the methods and drugs used at this clinic in the pain management of pediatric oncological patients.

## Materials and methods

Ethics committee

Approval for the study was obtained from the Mersin University Clinical Research Ethics Committee (issue: 2020/323, date: 29/4/2020).

Study design

Patients under 18 years of age diagnosed with cancer and treated for pain in the gastroenterology clinic between 2002 and 2020 were included in this study. Patients over 18 years of age and those missing algology clinic follow-up data were excluded from the study. We evaluated 93 patients and excluded three patients who met the exclusion criteria. The demographic data, follow-up period, diagnosis and stage of cancer, cause and location of pain, systems involved, duration and intensity of pain, analgesic and adjuvant drugs prescribed, routes and duration of drug administration, complications, interventional procedures if performed, “pain intensity” scores before and after treatment, and daily and total analgesic consumption by patients were documented from algology hard copy follow-up records.

Principles of pain management

Pediatric oncological outpatients who are admitted to or present to our clinic are evaluated by an algologist with physical examination, laboratory values, and imaging methods. According to the definition of cause and severity of pain, the choice of treatment and method is determined on the basis of the WHO’s analgesic ladder principle. The WHO's analgesic ladder is used to organize analgesia treatment for pediatric cancer patients:

WHO step 1: Paracetamol and non-steroidal anti-inflammatory drugs (NSAIDs) for mild pain

WHO step 2: Weak opioids, paracetamol, and NSAIDs for mild-to-moderate pain

WHO step 3: Potent opioids, paracetamol, and NSAIDs for severe pain

We prefer to use codeine and tramadol as weak opioids, and morphine, fentanyl, and oxycodone as potent opioids. Tramadol and the WHO step 2 were not used under the age of 12 after 2018, when the Ministry of Health of our country restricted its use in children. Adjuvant medications might be added for specific pain subtypes (Table 1). A sufficient dose and an appropriate pharmacologic formulation are chosen to enable children and their families to sleep undisturbed throughout the night without waking up with pain.

**Table 1:** Adjuvant drugs administered according to the source of pain.

Types	Drugs	Indications
Anticonvulsants	Pregabalin	Neuropathic pain
	Gabapentin	
	Tegretol	
Corticosteroid	Dexamethasone	Bone metastasis
Antidepressant	Amitriptyline	Neuropathic pain
Benzodiazepine	Alprazolam	Anxiety, insomnia

Interventional pain management applications (regional peripheral blocks or epidural catheterization) were used when indicated. Bupivacaine or morphine was administered when necessary.

Follow-up

At least twice a day, bedside visits were conducted to patients who were inpatients. Patients are followed until their pain levels drop to an acceptable level. Outpatients are called for control on the next day and third day when a new medication is started. Medication adjustments and controls are made every 10 days.

Pain measurement

Different evaluation methods were adopted because the recognition and compliance of children at different ages are distinct. The Revised Faces Pain Scale, visual analog scale (VAS), and numerical rating scale (NRS) are used as pain severity scales according to pediatric age groups in diagnosis and follow-ups at our clinic. In addition, the revised faces pain scale is included in the follow-up charts for pain monitoring in all pediatric services. The scores of the scales used are between 0 and 10. The degree of pain was scored as “0,” representing no pain, and “10,” representing the most severe pain.

For our study, the results of the scales were defined as the “pain score.” Pain in the form of tingling, burning, and stabbing was evaluated as “neuropathic pain.”

Statistical analysis

The data for the statistical analysis were entered using SPSS Version 24 (Statistical Package for Social Sciences) application. The E-PICOS calculator was also used to do calculations in accordance with the MedicReS Good Biostatistical Practice recommendations. For categorical data, descriptive statistics were used, and percentages were used to explain frequency calculations. The chi-square method was used to evaluate categorical variables. A p-value of 0.05 was used to determine statistical significance.

## Results

Of the 90 children, 45.6% (n = 41) were female, and the mean age was 11.4±4.1 (range: 2-17 years). The mean weight was 37.9±17.5 kg (range: 10-86 kg), and 76.7% (n = 69) of the patients were hospitalized pediatric patients. At admission, stage 4 cancer was present in 27 (87.1%) of the 31 individuals for whom staging was available.

**Figure 1:**
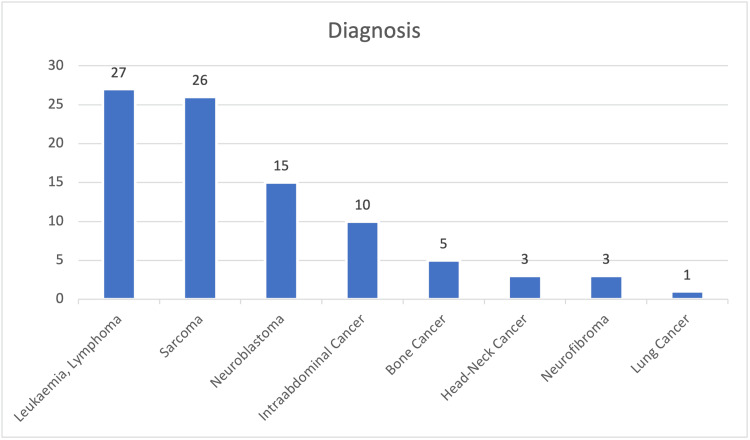
Oncological diagnosis distribution of our patients.

Leukemia and lymphoma were the most frequently diagnosed malignancies in children, occurring at a rate of 30% (n = 27) (Figure 1).

The causes of pain in children were neoplasms in 81.2% (n = 73), surgeries in 8.9% (n = 8), radiation in 4.4% (n = 4), and inflammation in 2.2% (n = 2). The localizations of pain at the first examination of the children are shown in Table 2.

**Table 2:** Distribution of pain localizations in children's first examination

Pain localizations	n (%)
Lower extremity	25 (27.8)
Abdominal	24 (26.7)
Lumbar – back	11 (12.2)
Head-face-mouth	11 (12.2)
Upper extremity – shoulder	5 (5.6)
Thoracic	5 (5.6)
Anal – perianal	3 (3.3)
More than three regions	3 (3.3)
Pelvic	2 (2.2)
Cervical	1 (1.1)

The examination of the duration and severity of pain at admission revealed that 27.8% (n = 25) of the patients had mild to moderate pain, 45.6% (n = 41) had severe pain that kept going for less than a month, 10.0% had severe pain that persisted for one to three months, and 16.7% (n = 15) had severe pain that went on for longer than three months.

While the mean initial pain scores of the children were 5.2±1.7 (min-max: 2-9), it decreased to 1.5±0.7 (min-max 1-4) with a significant difference (p<0.001). It was determined that seven (7.8%) of 36 children used a combination of paracetamol and ibuprofen. Detailed use of non-opioid analgesics is shown in Table 3.

**Table 3:** Distribution of use by types of non-opioid drugs. *Those who did not use non-opioid drugs.

Non-opioid analgesics	n (%)
Paracetamol	36 (40.0)
Ibuprofen	10 (11.1)
Naproxen	7 (7.8)
Paracetamol + ibuprofen	7 (7.8)
Diclofenac	1 (1.1)
None used^*^	29 (32.2)

Of the 90 patients, 81 (90%) used opioids and 26 (28.9%) used dual opioids. Nine (10%) patients had not received opioid treatment. Three (3.3%) patients with a starting pain score of 3 had taken paracetamol. The patient who required the most time-consuming care had an intracranial tumor treated with a fentanyl patch for 22 months. Pethidine was applied by the pediatric surgeon for postoperative analgesia. The opioids used are detailed in Table 4.

**Table 4:** Opioid drugs used Note: Since 26 of 90 patients used dual opioids, the percentage values are given according to 90 patients. *Those who did not use any opioids.

Opioids	n (%)	Mean daily dosage (min-max), mg/day	Duration of treatment days, n (min-max)
Weak opioids			
Tramadol	47 (52.2)	129.0±97.9 (12-380)	39.7 (1-366)
Codeine	2 (2.2)	90 (90-90)	
Potent opioids			
Morphine	39 (43.3)	14.8±11.3 (1-52)	18.5 (1-187)
Fentanyl patch	12 (13.3)	33.2±21.6 (12-75)	144.3 (4-664)
Oxycodone	1(1.1)	50 (50-50)	50 (50-50)
Pethidine	6 (6.7)	61.5±47.8 (15-120)	6.3 (1-17)
None used^*^	9 (10)		

When we listed the diagnoses of individuals using adjuvant drugs (pregabalin, gabapentin, tegretol, and amitriptyline) for neuropathic pain (n = 17, 18.8%), it was found that nine had sarcoma, three had neuroblastoma, three had bone tumor, and two had leukemia-lymphoma. The frequency of adjuvant drug use is given in Table 5.

**Table 5:** Distribution of use by types of adjuvant drugs. *Those who did not use any adjuvant drugs.

Adjuvant drugs	n (%)
Pregabalin	10 (11.1)
Gabapentin	3 (3.3)
Tegretol	2 (2.2)
Dexamethasone	5 (5.6)
Alprazolam	2 (2.2)
Amitriptyline	2 (2.2)
None used^*^	66 (73.3)

Epidural catheterization was performed in six children. Initial pain scores of those six patients were as follows: 8 in two patients, 7 in three patients, and 4 in one patient. Of the six children, four had musculoskeletal cancer, one had a gastrointestinal tumor, and one had a genitourinary system tumor. All of them had abdominal, perianal, and lower extremity pain.

Complications of pain management were nausea-vomiting in 4.4% (n = 4) and itching in 2.2% (n = 2). Complications were not recorded in 93.3% (n = 84) of children. Among children, cancer or its complications caused deaths of 30% (n = 27) of them.

## Discussion

The study results demonstrate that multimodal analgesia is a practical treatment for pediatric cancer-related pain. A patient was admitted with stage 4 and severe pain in the study population. With the potent opioids and interventional procedures, analgesia can be provided safely with close follow-up. Effective analgesia was provided compared to baseline treatment and end mean pain scores for all patients (5.2±1.7 and 1.5±0.7, respectively).

The most common childhood cancers are listed in the literature. Among these cancers, leukemia is responsible for 30%, brain tumors for 25%, lymphoma for 15%, neuroblastoma for 8%, intra-abdominal sarcoma for 15%, retinoblastoma for 3%, and liver tumors for 15% [9]. Except for brain tumors, the current study findings align with this distribution, possibly because individuals with brain tumors have a shorter life expectancy. Therefore, the number of patient admissions with brain tumors might be low.

Non-opioid drugs are commonly used to treat mild-to-moderate cancer pain and are recommended in the WHO cancer pain treatment ladder, alone or in combination with opioids [10]. Paracetamol or NSAIDs were used by 67.8% of the patients. Due to various contraindications, the remaining 32.2% either stopped using or discontinued the medication.

Mild opioids are often preferred for palliation of cancer pain. WHO’s analgesic ladder was used as a protocol in the pediatric cancer pain trial, which included 224 patients. The authors revealed that tramadol was the most frequently used weak opioid [11]. Tramadol was the opioid most frequently used in the clinic until 2018. For the last five years, weak opioid use in children under 12 years old was discontinued, in line with the recommendations by WHO and the Ministry of Health, but it is still frequently used for those over 12 years of age.

Opioids, such as morphine, fentanyl, and oxycodone, remain the gold standard of pharmacology for children in severe pain [12]. Moreover, transdermal fentanyl is an effective, safe, and well-tolerated treatment for pediatric cancer-related pain in opioid-naive patients with moderate-to-severe chronic pain [13]. In contrast, pethidine has limited use in pediatrics [14]. Analgesia with morphine was given to 43.3% (n = 39) of the patients. A fentanyl patch was used by 12 (13.3%) children from the study sample at a 12-75 mcg/h dosage. Pethidine was administered for only postoperative acute pain of patients under follow-up (6.7%, n = 6). The use of pethidine can be considered to be due to a lack of pain knowledge among physicians. Opioids were used in 90% of the pediatric cancer patients. Potent opioids can provide effective analgesia without serious side effects.

Cancer-related neuropathic pain is common and can be disease-related or related to the acute or chronic effects of cancer treatment [15]. According to Sen and Uzunhan, out of 160 patients monitored for childhood malignancies, 16% (n = 26) developed neuropathic pain [16]. No accepted pediatric dosing of pregabalin exists for neuropathic pain. The initial starting dose of 0.5 up to 6 mg/kg can be titrated in steps [17]. Pregabalin use was reported in one trial of 30 pediatric oncology patients with solid tumors or leukemia, with a dosage between 75 and 300 mg/day (5 mg/kg) [18]. For pediatric patients’ neuropathic pain, adjuvant medications were regularly used. After informing the patients of its adverse effects, permission to use pregabalin was obtained from the patient’s parents. We also used the dose range of 3 to 5 mg/kg/day, in line with the literature.

According to the WHO guidelines, steroids should be used to treat cancer pain in adults and adolescents experiencing visceral, neuropathic, or metastatic bone pain [19]. Notably, patients with bone metastasis (5.6%, n = 5) were administered dexamethasone. Due to its adverse effects, it should only be taken cautiously and in selected cases.

A multimodal approach to pain management is suggested to achieve adequate pain control, including potential interventional techniques [20]. In Mehl et al.’s study including 36 pediatric oncology patients, epidural and subcutaneous analgesia were compared, and it was shown that the epidural approach provided better analgesia [21]. Cuviello et al. investigated interventional approaches for pain control in pediatric oncology. They revealed that regional pain control interventions can effectively relieve pain related to pediatric cancer [22]. A study investigating epidural blocks in children with cancer suggested that a continuous catheter-delivered pain blockade contributes to analgesia and moderates opioid requirements. They found no bleeding, infectious, or neurological complications in these 10 children with epidural catheters [23]. Epidural catheterization was performed on six patients with lower extremity, perianal, and abdominal pain. None of the patients experienced complications; thus, this option should be considered for pediatric patients.

Opioid side effects might occur in approximately 14% of pediatric cancer patients. Opioid side effects, such as constipation, sedation, pruritis, and nausea, are often unavoidable [24]. It has been noted that fentanyl may cause coughing [25]. Additionally, Saadat et al. examined the prescriptions of pediatric opioid analgesics in a pediatric hospital. They concluded that restricting weak opioids increased the prescription of potent opioids, which could play a role in the overall rise in pediatric hospitalizations related to opioids [26]. Mild side effects were experienced by only 7.4% of the patients who received opioid therapy. In the study sample and the literature, severe side effects, such as sedation and respiratory depression, were rarely observed with a close follow-up. Closely monitored patients rarely have opiate side effects, which are held responsible for the ineffective management of pediatric oncological pain.

Limitations

The key limitation of our study was its retrospective design. Although our clinic has a process for treating children with cancer, each patient is assessed individually before a treatment choice is determined. This is the study's standardization limitation. The single-center nature of our study was our limitation in terms of the number of patients. Another one of our limitations was the variation in age groups, oncological diagnoses, and pain management in our patient sample. Due to our large age range, one of our limitations was that the pain assessment scores we used varied. Instead of reporting the doses we used for breakthrough pain, we had to show the total opioid consumption. The current study includes approximately 20 years of patient data. An additional limitation of the study is the limited number of pain interventions administered within this timeframe. Interventional pain management has come a long way in this process.

## Conclusions

The pain intensity experienced by pediatric cancer patients significantly affects their and their family’s quality of life. In this study, participants were not referred to the pain clinic until they reached stage 4 cancer. Opiophobia may be prevented partly because opioid-related side effects linked to pediatric pain management occur considerably less frequently than previously believed. Studies examining the multidisciplinary approach to pain management must be conducted, as effective pain management is crucial to improving quality of life.
